# Mediating and Moderating Effects of Uncertainty on the Relationship between Family Function, Self-Care, and Depression among Blood Cancer Survivors

**DOI:** 10.3390/bs14030170

**Published:** 2024-02-23

**Authors:** Hyun-E Yeom, Da-Som Park

**Affiliations:** 1 Department of Nursing, Chungnam National University, 266 Munhwaro, Junggu, Daejeon 35015, Republic of Korea; 2 Chungnam National University Hospital, 282 Munhwa-ro, Junggu, Daejeon 35015, Republic of Korea; ektha0617@cnuh.co.kr

**Keywords:** cancer survivors, depression, family support, self-care, uncertainty

## Abstract

Uncertainty in cancer survivorship poses a substantial challenge to survivors’ coping mechanisms and psychological well-being. This study investigated the intricate interplay among family function, uncertainty, self-care, and depression in this context, with a primary focus on discerning the mediating and moderating roles of uncertainty in the relationship between family function, self-care, and depression among blood cancer survivors. Cross-sectional data from 147 survivors in South Korea underwent analysis using descriptive statistics, Pearson’s correlations, and the PROCESS macro in SPSS version 26.0. The results revealed that family function significantly predicted both self-care and depression. Notably, uncertainty mediated the relationship between family function and these outcomes. Furthermore, the impact of family function on depression was moderated by uncertainty, indicating a relatively weaker association in survivors facing higher uncertainty levels. This study contributes valuable insights by elucidating the role of uncertainty in regulating how family function influences self-care and depression among survivors of blood cancer. It emphasizes the critical need to enhance family function and alleviate uncertainty for the improved adjustment of cancer survivorship. The findings underscore the importance of targeted support for individuals grappling with different levels of uncertainty, aiming to prevent or mitigate depressive symptoms through the reinforcement of family function.

## 1. Introduction

The World Health Organization recognizes cancer as a notable chronic condition that demands ongoing and vigilant self-care [1,2,3]. Blood cancer is a type of malignancy originating in blood-forming tissues and bone marrow [4]. It encompasses various types, with leukemia, lymphoma, and myeloma being predominant, along with myelodysplastic syndromes and myeloproliferative neoplasms [4]. Recent advances in therapeutic techniques, such as hematopoietic stem cell transplantation, targeted therapies, and high-dose chemotherapy, have significantly increased the survival rates of patients with blood cancer [4,5]. The five-year survival rates are 53.4%, 64.0%, and 84.5% for leukemia, non-Hodgkin lymphoma, and Hodgkin lymphoma, respectively [5]. However, unlike other cancers that are restricted to specific tissues or periods, blood cancer disrupts the normal functioning of hematopoiesis, blood cells, or the lymphatic system. This pervasive pathology impacts one’s overall health [4,5]. Hence, throughout all phases of diagnosis, treatment, and ongoing prognosis monitoring, consistent self-care is crucial for survivors of blood cancer [2,4]. Comprehensive self-care is essential and involves proactive prevention against the risk of infection and bleeding, and maintaining a balanced nutritional intake [3,4].

Cancer survivors face uncertainty, a prevalent psycho-cognitive issue, at any stage, including their diagnosis, treatment, and transition to follow-up care [2,6]. Abundant cancer research argues that uncertainty is associated with various adverse physical and psychological outcomes, such as increased symptom severity, fear, emotional distress, and feelings of losing control [7,8,9]. Also, uncertainty affected survivors’ engagement in self-care, aided them in overcoming the challenges of their diagnosis and treatment, and influenced the lifestyle adjustments they were required to adapt [10,11]. Many empirical studies have shown that uncertainty was associated with knowledge of the diagnosis, treatment, and the prognosis of diseases, as well as psychosocial factors, such as anxiety and support, throughout the process [12,13,14]. Survivors of blood cancer face uncertainty regarding the ambiguity and unpredictability stemming from vague symptoms across the entire body system, dynamic treatments, and long-term prognosis [2,4,8]. Despite these unique aspects, research has not comprehensively identified the influence of uncertainty on self-care or psychological distress, such as depression and anxiety, among survivors of blood cancer nor considered its association with other psychosocial factors.

It is unequivocal that individuals who have survived cancer seek support to navigate the physical and psychological challenges inherent in the therapeutic interventions and transitional phases they undergo [15,16]. Supportive family functioning is crucial in establishing a beneficial living environment for overcoming difficulties [17,18]. Support involves practical assistance with transportation to medical appointments, regular exercise, a balanced diet, and the avoidance of harmful habits, all of which contribute to the overall self-care routine and help maintain a sense of normalcy [19,20]. In addition, supportive family functioning involves emotional support, information sharing, adaptability, and shared decision-making based on effective communication [18,19,20]. Numerous studies have emphasized the significance of active interaction with family members, which provides a chance to express an open dialogue regarding the challenges and concerns related to the cancer experience, including uncertainty and depression [19,21]. Against the continuous challenging processes of cancer survivorship, supportive family functioning can enhance adaptive coping behavior, which is essential for managing stress, uncertainty, and the emotional toll.

An expanding body of theory-based empirical cancer studies highlights the pivotal role of family function as an integral component of social support in shaping individuals’ perceptions and managing uncertainty associated with their illness [17,18]. Within this context, cancer survivors exhibit the capability to develop both physical and psychosocial coping mechanisms to navigate uncertainty, recognizing the intrapersonal and family-based power to alleviate psychological distress, including depression, linked to uncertainty [9,15,17,18,22]. The existing literature in the realm of cancer research has found that a well-functioning family contributes significantly to cultivating adaptive coping strategies, thereby enhancing survivors’ capacity to navigate and manage uncertainties [21]. However, as emphasized in previous investigations, uncertainty persists from the conclusion of initial cancer treatment throughout survivors’ lifetimes, involving both survivors and their families [2,14,22,23]. Consequently, uncertainty becomes intricately intertwined with the mechanisms through which family function exerts influence on self-care and depression among individuals grappling with cancer survivorship.

Given the pivotal roles of uncertainty and family function in influencing coping behaviors and health outcomes, it is essential to comprehend their interactive dynamics concerning self-care practices and depressive symptoms during survivorship. Therefore, this study seeks to explore the mediating and moderating roles of uncertainty in the relationship between family function and other variables, including self-care and depression, among individuals who have survived blood cancer.

## 2. Materials and Methods

### 2.1. Design

This cross-sectional descriptive study explored how family function and uncertainty are associated with behavioral self-care and psychological distress among survivors of blood cancer.

### 2.2. Setting 

The recruitment of participants and data collection for this research were carried out following the STROBE guidelines pertinent to observational cross-sectional studies. The study protocol, including its aims and all associated procedures, received approval from the affiliated university hospital’s institutional review board (Approval number: CNUH 2021-06-080-006). 

Data were collected at a university hospital in Daejeon, South Korea. This phase spanned the period from 28 October 2021 to 28 February 2022. Prospective participants were provided with a comprehensive explanation of the study’s objectives and procedures. There was a clear communication of the potential risks and benefits of participation. Ethical considerations prioritizing the welfare of the participants were strictly adhered to, including stressing the importance of their autonomy in deciding to withdraw from the study at any time. A firm commitment was made to ensure that data would be handled anonymously, kept confidential, and used solely for the purposes of the research.

### 2.3. Participants

Participants for the study were recruited via convenience sampling from both outpatient and inpatient facilities at a university hospital. The inclusion criteria specified that candidates must be adults aged 19 years or older with a diagnosis of hematologic malignancies and must be actively undergoing treatment as inpatients or participating in outpatient follow-up care. Additionally, to qualify for inclusion, individuals were required to demonstrate no cognitive impairments, possess a clear and comprehensive understanding of the study’s objectives, and voluntarily agree to provide informed consent. Exclusion criteria were established to omit individuals receiving palliative care in the end-of-life stage or those who had been referred to hospice care. Initially, 160 individuals met the selection criteria. However, 6 participants failed to complete the questionnaire, and 7 were excluded due to insufficient responses, which resulted in 147 participants.

### 2.4. Sample Size

The adequacy of the sample size was evaluated using the G*Power 3.1.9.7 program [24]. Based on a medium effect size (f^2^) of 0.15 for linear multiple regression analysis [25], a type I error probability (α) of 0.05, and seven predictors, the determined sample size of 147 was deemed adequate. It provided a statistical power of 92.8%.

### 2.5. Measures

#### 2.5.1. Family Function

We assessed the extent to which cancer survivors perceived how their family members were supportive of each other using the Family APGAR scale [26]. The original scale was validated as a reliable tool to capture individuals’ perceptions about family functioning in Korean populations within healthcare contexts [27]. This scale comprised five items that encompassed the levels of adaptation, partnership, growth, affection, and resolve that respondents perceived on a regular basis in daily interactions with family members. Each item was rated on a 3-point Likert scale (0 = almost never, 1 = occasionally, and 2 = almost always). A score of family function was computed by summing the responses to all items, which ranged from 0 to 10 points. Higher scores denoted better family functioning. Reliability in this study was Cronbach’s α = 0.92. 

#### 2.5.2. Uncertainty

Uncertainty was measured with a Korean version of Mishel’s Uncertainty in Illness Scale-Community form (MUIS-C) [28,29]. The MUIS-C has been applied as a valid measure to assess the level of uncertainty experienced by individuals living with chronic illness across diverse countries. The scale translated into Korean [29] has been validated as a reliable scale to measure the level of uncertainty related to the complexity of the medical information and the lack of clarity regarding the state of the illness and its treatment among Korean cancer survivors. The MUIS-C comprised two dimensions: ambiguity (11 items) and complexity (12 items). Responses were rated on a 5-point Likert scale that ranged from 1 (not at all) to 5 (very much so). Some items were reverse-scored, and the total score ranged from 23 to 115 points. Higher scores indicated greater levels of uncertainty. Reliability in the previous Korean population [29] and this study were Cronbach’s α = 0.85 and 0.84.

#### 2.5.3. Self-Care Behavior

The assessment of self-care behavior utilized a specially developed and validated instrument designed for Korean patients with hematologic malignancies [30]. This scale has been recognized as reliable and valid for evaluating the self-care practices pertinent to this population [30,31], encompassing general and specific behaviors critical for preventing complications linked to their condition and enhancing health outcomes. The scale categorizes self-care activities into three areas: infection management (eleven items), bleeding prevention (four items), and nutrition (four items), with each item assessed on a 4-point Likert scale from 1 (not performed at all) to 4 (always performed well). The score for self-care behaviors ranged from 19 to 76 points, and higher scores indicated a higher level of self-care performance. The original instrument [30] and this study exhibited a reliability coefficient of Cronbach’s α = 0.85 and 0.91.

#### 2.5.4. Depression

Depressive symptoms were evaluated utilizing the Patient Health Questionnaire-9 (PHQ-9), which is globally used to assess the severity of depressive symptoms and screen for depressive disorders in diverse healthcare settings [32]. The PHQ-9 was validated across Korean populations with various sociodemographic and medical conditions [33]. It comprised nine items that rated the severity of problems experienced over the previous two weeks as not at all (0-point), several days (1-point), more than half the days (2-point), and nearly every day (3-point). Higher scores indicated greater severity of depressive symptoms. The overall level of depression was categorized based on the summed scores: 0–4, 5–9, 10–19, and 20–27 points as none, mild, moderate, and severe depression, respectively. In this study, the internal consistency reliability was Cronbach’s α = 0.86.

#### 2.5.5. Sociodemographic and Cancer-Related Characteristics

Participants’ sociodemographic characteristics, such as age, gender, marital status, living arrangements, educational level, employment status, and family economic status, were investigated. Regarding blood cancer, specific characteristics, such as the number of hospitalizations, duration since cancer diagnosis, the type of treatment received for the cancer, and the occurrence of relapse, were examined.

### 2.6. Statistical Analysis

Collected data were analyzed via SPSS/WIN software (version 26.0; IBM Corp., Armonk, NY, USA). Preliminary analyses were performed to assess the data distribution, skewness, kurtosis, and the presence of outliers for the main study variables of family function, uncertainty, self-care behavior, and depressive symptoms. Descriptive statistics, which included mean (M), standard deviation (SD), minimum and maximum values, frequency, and proportion, were computed for all the study variables to assess the overall characteristics. The internal consistency of the scales that pertained to the main study variables was evaluated via Cronbach’s alpha coefficients.

Pearson’s correlation coefficients and independent t-tests were computed to identify the covariates from sociodemographic and cancer-related characteristics. Our hypothesized model, which incorporated moderation and mediation effects, was assessed using the bootstrap method of PROCESS macro for SPSS [34]. We employed a bootstrapping technique that involved resampling numerous small samples, which mitigated the risk of type 1 errors and enhanced statistical power [34]. The analysis entailed the effect of the mediator (i.e., uncertainty) and moderator (i.e., uncertainty) on the relationship between the independent (i.e., family function) and dependent variables (i.e., self-care and depression). For the mediation effect of uncertainty, we utilized model 4, which tests an indirect effect of the independent variable (i.e., family function) on each of the dependent variables (i.e., self-care and depression) through the mediator (i.e., uncertainty). For the moderation effect of uncertainty, we utilized model 1, which tests an interaction effect of the independent variable (i.e., family function) with a potential moderator (i.e., uncertainty) on dependent variables (i.e., self-care and depression).

We utilized 5000 bootstrap samples to evaluate the significance of the mediation effect based on a 95% confidence interval (CI). If the CI does not include zero, the indirect effect is considered statistically significant, which means that a considerable mediation effect exists.

## 3. Results

### 3.1. Participants’ Descriptive Characteristics

Table 1 summarizes the participants’ sociodemographic and cancer-related characteristics. Their ages ranged from 25 to 85 years, with a mean of 59.87 (SD = 14.44). The sex distribution revealed a relatively higher proportion of males, comprising 82 individuals (55.8%), compared to 65 females (44.2%). Most were married (79.6%, *n* = 117) or cohabiting families (83.0%, *n* = 122). Furthermore, spouses were the primary caregivers in 52.4% of the cases (*n* = 77). Educational attainment varied as 52 (35.4%), 41 (27.9%), and 54 (36.7%) had completed middle school or lower, graduated from high school, and attained college degrees or higher, respectively. Of the respondents, 56.5% (*n* = 83) reported unemployment, and 53.7% (*n* = 79) reported that their monthly household incomes exceeded 2 million won.

Participants experienced an average of 2.03 (SD = 1.78) hospitalizations in the past year. The most common diagnosis was acute leukemia (49.0%, *n* = 72), followed by myelodysplastic syndrome (21.8%, *n* = 32) and malignant lymphoma (11.6%, *n* = 17). The time since initial diagnosis averaged 23.28 (SD = 26.33) months, and 47.6% (*n* = 70) had a duration of less than 12 months. Treatment modalities varied, and 49.7% (*n* = 73) received only chemotherapy, while 36.0% (*n* = 53) underwent hematopoietic stem cell transplantation. Most respondents (91.8%, *n* = 135) were in a non-recurrent state.

### 3.2. Differences in Self-Care and Depression According to the Sociodemographic and Cancer-Related Characteristics

We investigated the associations of sociodemographic and cancer-related characteristics with self-care and depression to identify covariates, employing independent t-tests and Pearson’s correlation coefficients. Significant associations were observed between self-care and several characteristics: age (r = 0.29, *p* < 0.001), education level (t = −5.04, *p* < 0.001), household income (t = −2.74, *p* = 0.007), and hospitalization status (t = 2.95, *p* = 0.004). Additionally, a significant relationship was found between depression and hospitalization status (t = −2.79, *p* = 0.006). Any other significant differences in self-care and depression according to different characteristics were not identified. Consequently, these characteristics were selected as covariates for further analysis.

### 3.3. Reciprocal Relationships between the Main Variables

Table 2 presents the interrelationships among uncertainty, family function, self-care, and depression. Uncertainty demonstrated a positive correlation with depression (r = 0.37, *p* = 0.000). Meanwhile, family function exhibited associations with self-care (r = 0.40, *p* = 0.000) and depression (r = −0.29, *p* = 0.000). Additionally, a negative correlation was observed between self-care and depression (r = −0.27, *p* = 0.000).

### 3.4. Indirect Impact of Family Function on Self-Care and Depression through Uncertainty

Table 3 illustrates the impact of family function and uncertainty on self-care and depression. Following adjustments for covariates that included age, sex, education level, family income, and duration since the initial cancer diagnosis, both family function and uncertainty remained significant predictors of self-care and depression. Also, notable indirect effects of family function were observed on both self-care (effect = 0.04; SE = 0.03; 95%CI = 0.015, 0.104) and depression (index = −0.08; SE = 0.04; 95% CI = −0.182, −0.009) through the mediating factor of uncertainty. The 95% CIs in both results do not include zero, indicating that there are significant indirect effects of family function on self-care and depression through uncertainty. The findings mean that uncertainty played a mediating role in transmitting the impact of family function to both self-care and depression.

### 3.5. Difference in the Impact of Family Function on Self-Care Behavior and Depression According to the Level of Uncertainty

Our analysis revealed a significant interaction effect (B = 0.38; SE = 0.18; *p* = 0.04) between family function and uncertainty in predicting depression, as shown in Table 3. The finding underscored the moderating role of uncertainty in the association between family function and depression. Conversely, our investigation indicated that the impact of family function on self-care was independent of uncertainty.

**Figure 1:**
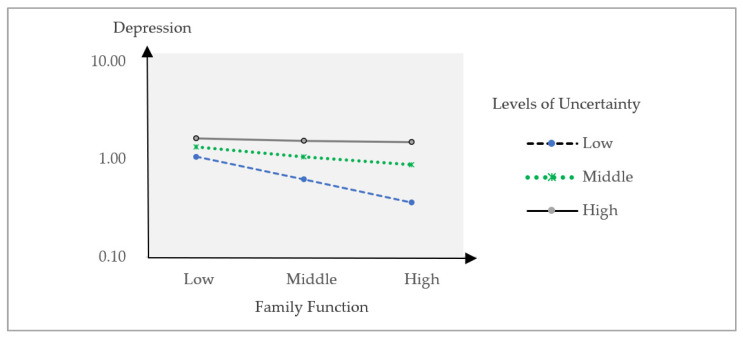
Comparative relationships between family function and depression according to the level of uncertainty.

Figure 1 compares the relationship between family function and depression across different uncertainty levels. Notably, the association was markedly stronger among participants who reported relatively lower levels of uncertainty, highlighting the pivotal role of family function in influencing depression, even when individuals had lower uncertainty.

## 4. Discussion

Despite advanced therapeutic techniques and increased survival rates, survivors of blood cancer face uncertainty from their initial diagnosis until the transition to survivorship. Considering the pivotal role of cancer-related uncertainty, this study identified its mediating and moderating role in the influence of family function on both self-care and depression.

A notable finding of the present study is the demonstration that family function influences self-care and depression through the mediating mechanism of uncertainty. These findings align with the established theoretical propositions and empirical evidence suggesting that family function can mitigate or exacerbate illness-related uncertainty [17,18]. Additionally, in accordance with prior evidence regarding uncertainty, our study underscores the impediment posed by uncertainty to self-care, concurrently elevating the risk of psychological distress [7,14]. Identifying uncertainty as a mediating factor is particularly noteworthy as it bridges the existing gaps in our understanding of the associations between family function, uncertainty, and outcomes related to self-care and depression. Consistent with theoretical and empirical research on various cancer survivors [15,17], our findings highlight the capacity of improved family dynamics to support self-care practices and reduce depressive symptoms.

Moreover, the present study provides evidence establishing that these relationships are closely linked to the uncertainty experienced by individuals within the family context. It aligns with prior research emphasizing the crucial roles of both family interaction and uncertainty in influencing self-care and depression [15,19,20]. Further, the current study contributes significantly by elucidating the role of uncertainty, which is partly contingent on family function, in shaping the interplay between family function, self-care, and depression.

Another critical revelation pertains to the moderating role of uncertainty in the relationship between family function and depression. The impact of family function on depression exhibited variations based on the level of uncertainty, revealing a more robust association among survivors with relatively low levels of uncertainty. The finding suggests that the impact of family function on depression is more pronounced when survivors experience lower levels of uncertainty. Conversely, when survivors confront considerable uncertainty, the influence of family function on depression diminishes. Therefore, this finding implies a relatively potent role of uncertainty in affecting depression, extending beyond the influence of family function.

This discovery also underscores the crucial role of supportive family interaction in influencing depression among cancer survivors, remarkably, even in those with lower levels of uncertainty. In line with empirical research on survivors across various types of cancer and stages [17,18,35,36], this study recognizes family function as a crucial factor associated with depression among survivors. Furthermore, the finding emphasizes the importance of prioritizing the reinforcement of family function to prevent and alleviate depression, particularly among those with lower levels of uncertainty.

On the contrary, given the myriad psychosocial and physical challenges encountered during cancer survivorship, both survivors and their families are compelled to adapt to a diverse range of stressors impacting family dynamics. Empirical studies [2,6,14] indicate that illness-related uncertainty extends beyond patients and affects family members. Consequently, in the presence of concurrent uncertainty within the family, the influence of family function on depression becomes more pronounced when perceived uncertainty is low. This tendency is attributed to the potentially limited support from family function in situations of higher uncertainty, thereby intensifying the challenges associated with depression.

While this study offers valuable insights, it is essential to acknowledge its limitations. Firstly, the current study collected data via self-administered surveys. Although the approach is common in socio-behavioral research, potential biases such as response and social desirability bias need to be considered [37]. Secondly, the participants were recruited by convenience sampling from a university hospital. This may impede the generalizability of the findings even though we included a diverse range of participants regarding outpatient and inpatient statuses, treatment histories, and sociodemographic profiles. Additionally, the cross-sectional design of the study, focused exclusively on blood cancer survivors, constrains our ability to make causal inferences about the impact of family function and uncertainty on survivors of other types of cancer. Considering the rising global incidence of cancer and the increase in survival rates, future studies should aim to encompass a wider variety of cancer survivors to broaden the applicability and significance of the research findings.

This study, despite its limitations, enriches our comprehensive and specific understanding of the role of uncertainty in cancer survivorship for guiding effective coping behaviors and enhancing psychological well-being among survivors. Notably, compared to other cancer types, the duration of treatment and recovery for blood cancer survivors exhibits substantial variability, ranging from a few weeks to several years, contingent upon the cancer subtype and the patient’s response. The average post-diagnosis period for survivors in this study was 24 months, indicating a transition to a chronic rather than an acute phase. This revelation highlights the persistent nature of uncertainty among cancer survivors, which is not ephemeral but endures over the long term, profoundly influencing self-care practices and depressive symptoms. Consequently, this study advocates for a thorough clinical evaluation of the uncertainty blood cancer survivors face, pinpointing it as a crucial step towards ameliorating their quality of life.

## 5. Conclusions

The dynamics of interactive family function and the uncertainty experienced by survivors of blood cancer were identified as pivotal factors influencing their behavioral self-care and psychological well-being. With a specific focus on the enduring impact of uncertainty during survivorship, this study revealed that uncertainty served as both a mediator and moderator, shaping the relationship between family function, self-care, and depression. The impact of family function on self-care and depression was partially mediated by uncertainty, illustrating the close intertwining of uncertainty with family function. Furthermore, the association between family function and depression varied based on the level of uncertainty, exhibiting a more pronounced association with lower uncertainty. Recognizing the intricate interplay between family function and uncertainty, both of which influence self-care and mental health, is crucial for healthcare professionals providing care to survivors of blood cancer. Tailoring interventions to strengthen family support and address uncertainty could significantly enhance self-care practices and overall well-being within this population.

## Figures and Tables

**Table 1:** Sociodemographic and cancer-related characteristics of the participants (N = 147).

Characteristics	Categories	M ± SD or n (%)	Range
Age		59.87 ± 14.44	25–78
Gender	Female	65 (44.2)	
	Male	82 (55.8)	
Education	Middle-school graduation or below	52 (35.4)	
	Highschool graduates or above	95 (64.6)	
Marital status	Married	117 (79.6)	
	Single, divorced, or others	30 (20.4)	
Employment status	Currently employed	64 (43.5)	
	Not employed	83 (56.5)	
Monthly household income	<2,000,000	68 (46.3)	
(Korean Won)	≥2,000,000	79 (53.7)	
Hematologic malignancy	Acute leukemia	72 (49.0)	
	Myelodysplastic syndrome	32 (21.8)	
	Malignant lymphoma	17 (11.6)	
	Aplastic anemia	11 (7.5)	
	Chronic leukemia	8 (5.4)	
	Multiple myeloma	7 (4.7)	
Primary caregiver	Spouse	77 (52.4)	
	Family other than spouse	29 (19.7)	
	Professional caregiver	15 (10.2)	
	None	26 (17.7)	
Hospitalization status	Inpatient	73 (49.7)	
	Outpatient	74 (50.3)	
Duration since a cancer diagnosis (months)		23.28 ± 26.33	2–116
Frequency of hospitalization (year)		2.03 ± 1.78	0–7
Recurrence	Yes	12 (8.2)	
	No	135 (91.8)	
Treatment modality	HSCT ^§^	53 (36.0)	
	Only chemotherapy	73 (49.7)	
	Others ^†^	21 (14.3)	

Note. ^§^ HSCT = Hematopoietic stem cell transplantation; ^†^ Others = No experience in medical treatment or currently treated with transfusion and immuno-suppressive therapy except for HSCT or chemotherapy.

**Table 2:** Reciprocal correlations between the main study variables (N = 147).

			1	2	3
	Sum	M (SD)	r *(p)*	r *(p)*	r *(p)*
1. Uncertainty	72.49	2.46 (0.56)	-		
2. Family function	8.57	1.71 (0.44)	−0.13 (0.11)	-	
3. Self-Care	64.32	3.39 (0.48)	−0.10 (0.24)	0.40 (0.00)	-
4. Depression	5.81	0.64 (0.56)	0.37 (0.00)	−0.29 (0.00)	−0.27 (0.00)

**Table 3:** Mediation and moderation effects of uncertainty for the influence of family function on self-care and depression.

Predictors	Self-Care			Depression		
	B	SE	*t (p)*	B	SE	*t (p)*
Constant	2.93	0.24	12.27(0.00)	0.15	0.31	0.49 (0.63)
Family function	0.33	0.08	4.31 (0.00)	−0.26	0.10	−2.62 (0.01)
Uncertainty	−0.20	0.07	−2.96 (0.00)	0.40	0.09	4.54 (0.00)
Covariates						
Age	0.01	0.00	1.96 (0.05)	−0.00	0.01	−0.14 (0.89)
Education ^§1^	0.33	0.09	3.65 (0.00)	−0.24	0.12	−2.05 (0.04)
Family income ^§2^	0.06	0.08	0.84 (0.41)	−0.00	0.10	−0.04 (0.97)
Hospitalization ^§3^	−0.13	0.07	−1.90 (0.06)	0.12	0.09	1.40 (0.16)
Indirect effect of family function via uncertainty	Effect (SE)	LL 95% CI	UL 95% CI	B (SE)	LL 95% CI	UL 95% CI
	0.04 (0.03)	0.015	0.104	−0.08 (0.04)	−0.182	−0.009
**Predictors**	**Self-Care**			**Depression**		
	**B**	**SE**	***t*** ***(p)***	**B**	**SE**	***t*** ***(p)***
Constant	3.01	0.17	17.94 (0.00)	0.77	0.21	3.65 (0.00)
Family function	0.32	0.08	4.10 (0.00)	−0.30	0.10	−3.01 (0.00)
Uncertainty	−0.20	0.07	−2.97 (0.00)	−0.39	0.09	4.51 (0.00)
Family function x Uncertainty	0.08	0.15	0.54 (0.59)	0.38	0.18	2.08 (0.04)
Covariates						
Age	0.01	0.00	1.87 (0.06)	−0.00	0.00	−0.39 (0.70)
Education ^§1^	0.33	0.09	3.62 (0.00)	−0.25	0.12	−2.12 (0.04)
Family income ^§2^	0.07	0.08	0.86 (0.39)	0.01	0.10	0.06 (0.95)
Hospitalization ^§3^	−0.13	0.07	−1.94 (0.05)	0.10	0.09	1.19 (0.24)

Note. Bootstrap samples = 5000; ^§^ Dummy variable; ^1^ Reference = middle-school graduation or below; ^2^ Reference = monthly household income under 2,000,000 won; ^3^ Reference = outpatients.

## Data Availability

The data supporting the findings of this study are not publicly available since participants did not give written consent for their data to be shared publicly.
